# Supplementary material for: MetaRibo-Seq measures translation in microbiomes
Source: Nat Commun. 2020 Jun 29;11:3268. doi: 10.1038/s41467-020-17081-z (PMC7324362; doi:10.1038/s41467-020-17081-z)
Supplement: Supplementary file 10 — Supplementary Data 7 [file 41467_2020_17081_MOESM10_ESM.zip › File2/Confidence_VeryHigh_Taxonomy/232815_out.krona.html]

Javascript must be enabled to view this page.

members
magnitude
magnitudeUnassigned
count
unassigned
taxon
rank

232815\_out

29

29
superkingdom
2

phylum
976
1

1
class
200643

1
171549
order

family
815
1

1
816
genus

1

SRS148159\_contig\_number\_22694
species
1262739

256845
phylum
1

1932692

SRS077392\_contig\_number\_contig-100\_4728.128958
species
1

phylum
1239
27

186801
class
27

27
order
186802

31979
family
18

18
genus
1485

1262828

SRS015190\_contig\_number\_contig-100\_7491.49119SRS016495\_contig\_number\_contig-100\_25414.61046SRS018623\_contig\_number\_7331SRS019068\_contig\_number\_13575SRS049900\_contig\_number\_contig-100\_79.113876SRS050998\_contig\_number\_contig-100\_3302.53520SRS063985\_contig\_number\_30705SRS077552\_contig\_number\_18991SRS1041033\_contig\_number\_14698SRS143372\_contig\_number\_1657SRS144135\_contig\_number\_14918SRS144362\_contig\_number\_18860SRS144714\_contig\_number\_contig-100\_18319.99978SRS149181\_contig\_number\_712SRS893170\_contig\_number\_contig-100\_15087.15088
species
15

3

SRS013687\_contig\_number\_contig-100\_6685.127398SRS077502\_contig\_number\_12509SRS148721\_contig\_number\_contig-100\_35822.35822
species
1896990

1
186806
family

genus
1730
1

39485
species

SRS014855\_contig\_number\_11805
1

family
186803
8

8
1898203

SRS015431\_contig\_number\_75719SRS015854\_contig\_number\_10477SRS019496\_contig\_number\_contig-100\_9872.9873SRS049773\_contig\_number\_29371SRS077194\_contig\_number\_30299SRS142503\_contig\_number\_28619SRS143523\_contig\_number\_14779SRS148424\_contig\_number\_33796
species
